# The Extracellular Calcium-Sensing Receptor in the Intestine: Evidence for Regulation of Colonic Absorption, Secretion, Motility, and Immunity

**DOI:** 10.3389/fphys.2016.00245

**Published:** 2016-06-21

**Authors:** Lieqi Tang, Catherine Y. Cheng, Xiangrong Sun, Alexandra J. Pedicone, Mansour Mohamadzadeh, Sam X. Cheng

**Affiliations:** ^1^Department of Pediatrics, Gastroenterology, Hepatology, and Nutrition, University of FloridaGainesville, FL, USA; ^2^Department of Medicine, Center for Inflammation and Mucosal Immunology, University of FloridaGainesville, FL, USA

**Keywords:** intestinal barrier function, enteric nervous system, gut immunity, secretory diarrhea, motility, irritable bowel syndrome, inflammatory bowel disease, calcium-sensing receptor

## Abstract

Different from other epithelia, the intestinal epithelium has the complex task of providing a barrier impeding the entry of toxins, food antigens, and microbes, while at the same time allowing for the transfer of nutrients, electrolytes, water, and microbial metabolites. These molecules/organisms are transported either transcellularly, crossing the apical and basolateral membranes of enterocytes, or paracellularly, passing through the space between enterocytes. Accordingly, the intestinal epithelium can affect energy metabolism, fluid balance, as well as immune response and tolerance. To help accomplish these complex tasks, the intestinal epithelium has evolved many sensing receptor mechanisms. Yet, their roles and functions are only now beginning to be elucidated. This article explores one such sensing receptor mechanism, carried out by the extracellular calcium-sensing receptor (CaSR). In addition to its established function as a nutrient sensor, coordinating food digestion, nutrient absorption, and regulating energy metabolism, we present evidence for the emerging role of CaSR in the control of intestinal fluid homeostasis and immune balance. An additional role in the modulation of the enteric nerve activity and motility is also discussed. Clearly, CaSR has profound effects on many aspects of intestinal function. Nevertheless, more work is needed to fully understand all functions of CaSR in the intestine, including detailed mechanisms of action and specific pathways involved. Considering the essential roles CaSR plays in gastrointestinal physiology and immunology, research may lead to a translational opportunity for the development of novel therapies that are based on CaSR's unique property of using simple nutrients such as calcium, polyamines, and certain amino acids/oligopeptides as activators. It is possible that, through targeting of intestinal CaSR with a combination of specific nutrients, oral solutions that are both inexpensive and practical may be developed to help in conditioning the gut microenvironment and in maintaining digestive health.

## Introduction

The intestinal epithelium is faced with the complex task of providing a barrier impeding the entry of noxious substances and microbes, while concurrently allowing for nutrient and water absorption and secretion. The primary function of the gastrointestinal (GI) tract is to digest food and absorb nutrients. To aid in digestion, the GI tract secretes a large amount of fluid to mix the food components and to lubricate the surface of the lumen. It is estimated that in a 24-h period, an average of 7.0 L of digestive juices are secreted upon food ingestion. These include 1.5 L from the salivary glands, 2.5 L from the stomach, 0.5 L from the biliary system, 1.5 L from the pancreas, and 1.0 L from the intestine (Boron and Boupaep, 2008). Upon completion of digestion and extraction of nutrients, these secretions must be reabsorbed along with released nutrients while also ensuring that further post-digestive secretions do not occur. These processes are highly regulated and coordinated; failure to do so may result in diseases such as mal-digestion, mal-absorption, constipation, or diarrhea.

The mammalian gut is considered the largest immune organ in the body. It is estimated that 65–80% of the body's immune cells (e.g., macrophages, dendritic cells, T cells, and B cells) and over 90% of immunoglobulin-producing cells are found in the gut, residing in ~100,000 isolated lymphoid follicles in the sub-epithelium lamina propria layer of the mucosa (Brandtzaeg et al., 1989). Adjacent to the epithelium is the lumen of the intestine, where, in addition to food and nutrients, hundreds of trillions of microorganisms reside. These include microbes that benefit the host, as well as those that cause disease. Additionally, during food ingestion, foreign antigens are being continuously introduced into the GI tract, and these molecules may also pose threats to the body. Accordingly, the GI tract must maintain an intact epithelial barrier and reliable immunity. The GI tract has the innate ability to control the flow of nutrients across the epithelium where the nutrients are absorbed, while also restricting microbes and food antigens to the lumen. In this manner, the immune cells of the gut are prevented from over-activation, and consequently, no inflammation, allergic reaction, or hypersensitivity results.

Notably, the mammalian epithelium lining the GI tract is a specially adapted tissue equipped with sensing receptor mechanisms. These sensing receptors are constantly detecting and responding to changes in the composition of local nutrient milieus and microbial environment. This constant monitoring by receptors ensures that the gut absorbs and secretes according to the state of digestion and nutrient availability, and that it alters intestinal permeability and immunity in accordance with the status of flora. One such sensing mechanism is the extracellular calcium-sensing receptor (CaSR), a multifaceted heptahelical guanine nucleotide-binding protein (G protein)-coupled receptor (GPCR; Brown et al., 1993).

In this article, after briefly exploring the function of CaSR as a nutrient sensor along the mammalian GI tract, we discuss the emerging roles this receptor may play in regulating colonic secretion, absorption, motility, epithelial integrity, and immunity. The role of CaSR in the regulation of colonic epithelial proliferation/tumorigenesis, differentiation, and stem cell growth and renewal has been reviewed elsewhere (see references Whitfield, 2009; Ghevariya and Anand, 2011; Macleod, 2013a; Singh et al., 2013; Tennakoon et al., 2016; also in the article by Kallay and colleagues and the discussion by MacLeod, et al in this issue) and is therefore not discussed here, even though it is very important for maintenance of intestinal barrier integrity/immunity. A general approach that was used to verify the role of CaSR in the studies presented here is either pharmacological, using the specific CaSR inhibitors (e.g., NPS 2143, calhex 231) or activators (e.g., R568, cinacalcet), or genetic, by comparing the behavior difference between mice that lack CaSR *vs*. their wild type controls. Both global CaSR exon 5 null mice [e.g., *Casr*^−∕−^*PTH*^−∕−^ (Kos et al., 2003) and *Casr*^−∕−^*Gcm2*^−∕−^ (Tu et al., 2003)] and intestine-specific CaSR exon 7 null mice [e.g., ^villin^*Cre/Casr*^flox∕flox^ Rey O et al., 2012] are generated and successfully used. Double global CaSR knockout mice are used because deletion of the CaSR gene alone results in early death from the toxic effects of unregulated release of parathyroid hormone (PTH) from parathyroid chief cells as well as from the pathological effects of the consequent hypercalcemia (Ho et al., 1995). As a result, double knockouts with simultaneous ablation of additional PTH gene (as in *Casr*^−∕−^*PTH*^−∕−^) or gene that regulates PTH (e.g., Gcm2 as in *Casr*^−∕−^*Gcm2*^−∕−^) are generated to “rescue” the lethal CaSR-deficient phenotype.

## CaSR and nutrient-sensing

CaSR is a well-conserved ancient GPCR, originally cloned from bovine parathyroid glands (Brown et al., 1993) and then found in rat kidney (Riccardi et al., 1995). There, it acts as an extracellular calcium ion (${\text{Ca}}_{\text{o}}^{2+}$) sensor and provides a key negative feedback mechanism for ${\text{Ca}}_{\text{o}}^{2+}$ to regulate parathyroid hormone secretion and urinary ${\text{Ca}}_{\text{o}}^{2+}$ excretion, thereby maintaining systemic ${\text{Ca}}_{\text{o}}^{2+}$ homeostasis (Brown and MacLeod, 2001; Hofer and Brown, 2003). It was subsequently found in other tissues of diverse species that are not typically associated with ${\text{Ca}}_{\text{o}}^{2+}$ homeostasis, thereby suggesting that this receptor may subserve other roles and functions beyond systemic ${\text{Ca}}_{\text{o}}^{2+}$ homeostasis.

Importantly, CaSR is a member of the class C GPCR that uses nutrients as agonists. This receptor, therefore, not only senses ions, but may also recognize and respond to nutrients in the milieus. Like other members in this family of GPCRs, such as metabolic glutamate receptors, gamma amino butyrate B receptors, sweet, and umami taste receptors, and pheromone receptors, CaSR is structurally equipped with an unusually large extracellular domain (~50% of the receptor mass) called the Venus fly trap module. Studies suggested that this Venus fly trap domain is located outside of the cell and senses nutrients, specifically protein breakdown products [e.g., amino acids (Conigrave et al., 2000; Mun et al., 2004), peptides (Conigrave and Brown, 2006; Wang et al., 2006; Broadhead et al., 2011), and polyamines (Quinn et al., 1997)] as well as other environmental cues [e.g., ionic strength and pH (Quinn et al., 2004)]. Considering that these nutrients/conditions are routinely encountered by cells/tissues in the GI tract, this nutrient sensor may play crucial roles in GI physiology.

Indeed, CaSR has been widely detected in tissues and cell types in the GI tract and its accessory organs that are implicated in nutrient sensing and/or nutrient handling. These include the taste cells in the taste buds of the tongue, the gastrin-secreting G cells, and the cholecystokinin (CCK)-secreting K cells in the stomach and duodenum, the nutrient-absorbing villous cells in the small intestine, the short chain fatty acid (SCFA)-absorbing surface cells in the large intestine, and the enteric nervous system (ENS; see summary in Table 1). In these cells and tissues, CaSR may act as a nutrient sensor, monitoring, and coordinating digestion, secretion and absorption. For example, in the mouth, which is the beginning of the sensory portion of the gut, CaSR may allow the taste cells to chemo-sense bitter taste (calcium), and kokumi taste (γ-glutamyl peptides; Ohsu et al., 2010; Maruyama et al., 2012), thus facilitating food ingestion. In the digestive gut (i.e., the stomach and duodenum), this nutrient sensor may enable the gastrin cells and the CCK cells to detect the arrival of food, stimulate digestive secretions, and initiate postprandial food digestion. In support of this notion, the gastric G cells in wild type mice or cells were found to release gastrin upon activation by luminal calcium, phenylalanine, peptone, spermine, or the calcimimetic Cinacalcet (Ray et al., 1997), but not those G cells in CaSR-pharmacologically inhibited or genetically ablated (*Casr*^−∕−^*PTH*^−∕−^) mice (Feng et al., 2010). Similar observations were made in the intestinal I cells, which responded to luminal nutrients, calcium, phenylalanine, tryptophan and peptides, and secreted CCK only in wild type mice or cells, but not in CaSR-null (*Casr*^−∕−^*PTH*^−∕−^) mice (Liou et al., 2011) or CaSR activity-inhibited cells isolated from wild type mice (Wang et al., 2011). These findings point to the significance of CaSR in nutrient sensing in the gut.

**Table 1:** **CaSR and modulations of digestive functions**.

**Cell types that express**	**Activating nutrient(s)**	**Role and function**	**Type of evidence**	**References**
**CELLS THAT REGULATE INGESTION AND DIGESTION**				
**Tongue**				
Taste buds	${\text{Ca}}_{\text{o}}^{2+}$, γ-glutamyl peptide	↑bitter and kokumi taste perception	Pharmacological	Ohsu et al., 2010; Maruyama et al., 2012
**Esophagus**				
Epithelial cells (basal cells)		Inflammation-modulating (?)	Biochemical	Justinich et al., 2008; Abdulnour-Nakhoul et al., 2015
**Stomach**				
G cells (ap & bl)	${\text{Ca}}_{\text{o}}^{2+}$, Phe, spermine, Peptide, cinacalcet	↑gastrin 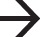 ↑gastric H+ production	Pharmacogenetic	Ray et al., 1997; Buchan et al., 2001
D cells			Biochemical	Haid et al., 2012; Adriaenssens et al., 2015
Ghrelin cells (ap & bl)	Phe, Ala, peptide	↑and ↓ghrelin secretion	Pharmacological	Engelstoft et al., 2013; Vancleef et al., 2015
Parietal cells (bl)	${\text{Ca}}_{\text{o}}^{2+}$, Mg2+, amino acid	↑gastric H+ secretion	Biochemical	Cheng et al., 1999; Caroppo et al., 2001; Geibel et al., 2001; Busque et al., 2005; Dufner et al., 2005
Mucous cells (ap & bl)	${\text{Ca}}_{\text{o}}^{2+}$	↑mucous secretion	Biochemical	Rutten et al., 1999; Gilster et al., 2004
**Duodenum**				
I cells (ap & bl)	${\text{Ca}}_{\text{o}}^{2+}$, Phe, Trp, peptide	↑CCK 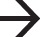 ↑pancreatic juice & bile	Pharmacogenetic	Sheinin et al., 2000; Hira et al., 2008; Nakajima et al., 2010, 2012; Liou et al., 2011; Wang et al., 2011
**Pancreas**				
Acinar cells		↑ secretion of pancreatic juice (?)	Biochemical	Bruce et al., 1999
Ductal cells (ap)	${\text{Ca}}_{\text{o}}^{2+}$	↑pancreatic fluid flow & solubility	Biochemical	Bruce et al., 1999
α & β cells of Islet	${\text{Ca}}_{\text{o}}^{2+}$, L-His	↑insulin 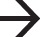 ↑nutrient utilization	Biochemical	Bruce et al., 1999; Squires et al., 2000; Komoto et al., 2003; Leech and Habener, 2003
**Liver**				
Hepatocytes	${\text{Ca}}_{\text{o}}^{2+}$, spermine	↑bile flow	Biochemical	Canaff et al., 2001
Cholangiocytes (?)				
**CELLS THAT REGULATE ABSORPTION AND SECRETION**				
**Small intestine**				
Villus cells (ap & bl)		↑absorption	Biochemical	Chattopadhyay et al., 1998
K cells	${\text{Ca}}_{\text{o}}^{2+}$, amino acid	↑GIP 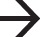 ↑insulin/nutrient utilization	Pharmacological	Mace et al., 2012
L cells	${\text{Ca}}_{\text{o}}^{2+}$, amino acid, peptide	↑GLP-1 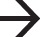 ↑insulin/nutrient utilization	Pharmacological	Leech and Habener, 2003; Mace et al., 2012; Diakogiannaki et al., 2013; Pais et al., 2016
**Colon**				
Surface & crypt cells (ap & bl)	${\text{Ca}}_{\text{o}}^{2+}$, polyamine, R568	↑absorption; ↓secretion	Pharmacogenetic	Chattopadhyay et al., 1998; Cheng et al., 2002; Cheng, 2012
		↑barrier function; ↑gut immunity	Pharmacogenetic	Jouret et al., 2013; Macleod, 2013b; Cheng et al., 2014
		↓proliferation; ↑differentiation	Pharmacogenetic	Chakrabarty et al., 2003; Rey O et al., 2012; Aggarwal et al., 2015
		↓colon cancer	Pharmacogenetic	Kallay et al., 2000; Sheinin et al., 2000; Singh and Chakrabarty, 2013; Aggarwal et al., 2015; Singh et al., 2015
**CELLS THAT REGULATE OTHER FUNCTIONS**				
Myofibroblasts	${\text{Ca}}_{\text{o}}^{2+}$	↑wnt5a and BMP-2 secretion	Biochemical	Peiris et al., 2007; Pacheco and Macleod, 2008
ENS	peptide, R568	↓secretion; ↓motility	Pharmacological	Chattopadhyay et al., 1998; Cheng, 2012; Muramatsu et al., 2014
Immune cells	${\text{Ca}}_{\text{o}}^{2+}$	↓inflammation	Biochemical	Kelly et al., 2011

*Ala, alanine; ap, apical membrane; bl, basolateral membrane; ${\text{Ca}}_{\text{o}}^{2+}$, extracellular calcium; CCK, cholecystokinin; ENS, enteric nervous system; GIP, glucose-dependent insulinotropic peptide; GLP-1, glucagon-like peptide-1; Phe, phenylalanine; PYY, peptide tyrosine tyrosine; Trp, tryptophan*.

In the absorptive gut (i.e., the jejunum and ileum) where dietary nutrients are fully released and extracted from ingested food, CaSR may function to inform the villus cells of the availability of nutrients, to activate absorption, and to provide a mechanism to signal to the ENS, coined “the brain of the gut,” to coordinate food delivery and gut motility in order to maximize nutrient absorption. In this latter part of the gut, CaSR has also been found to be present in a number of enteroendocrine cells, including the glucagon-like peptide-1 (GLP-1)-secreting L cells (Mace et al., 2012; Pais et al., 2016), the glucose-dependent insulinotropic peptide (GIP)-secreting K cells (Mace et al., 2012), and the insulin-secretion β cells of the pancreatic Islets of Langerhans (Leech and Habener, 2003). Since the main function of GLP-1 and GIP is to enhance glucose-induced insulin secretion from β cells, it is possible that CaSR may play a role in nutrient (glucose/SCFA) utilization and energy homeostasis, through regulating the postprandial secretion of GLP-1, GIP, and insulin. Indeed, oral or duodenal administration of CaSR peptide agonists to experimental animals reduced rapid elevation of plasma glucose in response to oral glucose challenge (Muramatsu et al., 2014). Further, evidence supporting the role of CaSR as a nutrient sensor and food metabolism regulator comes from the discovery that CaSR is found in tissues that regulate appetite and satiety. In addition to the duodenal I cells that secrete satiety-inducing CCK, there is evidence that gastric ghrelin cells, which secrete ghrelin (a hunger hormone), express CaSR, too (Engelstoft et al., 2013; Vancleef et al., 2015). Thus, CaSR may participate in the regulation of food intake. Taken together, it is tempting to speculate that modulations of intestinal CaSR expression and function by calcimimetics or nutritional receptor agonists may alter the behavior of food intake and energy metabolism thus providing a novel pathway for prevention and treatment of both obesity and type 2 diabetes mellitus.

## CaSR and intestinal absorption and secretion

**Figure 1:**
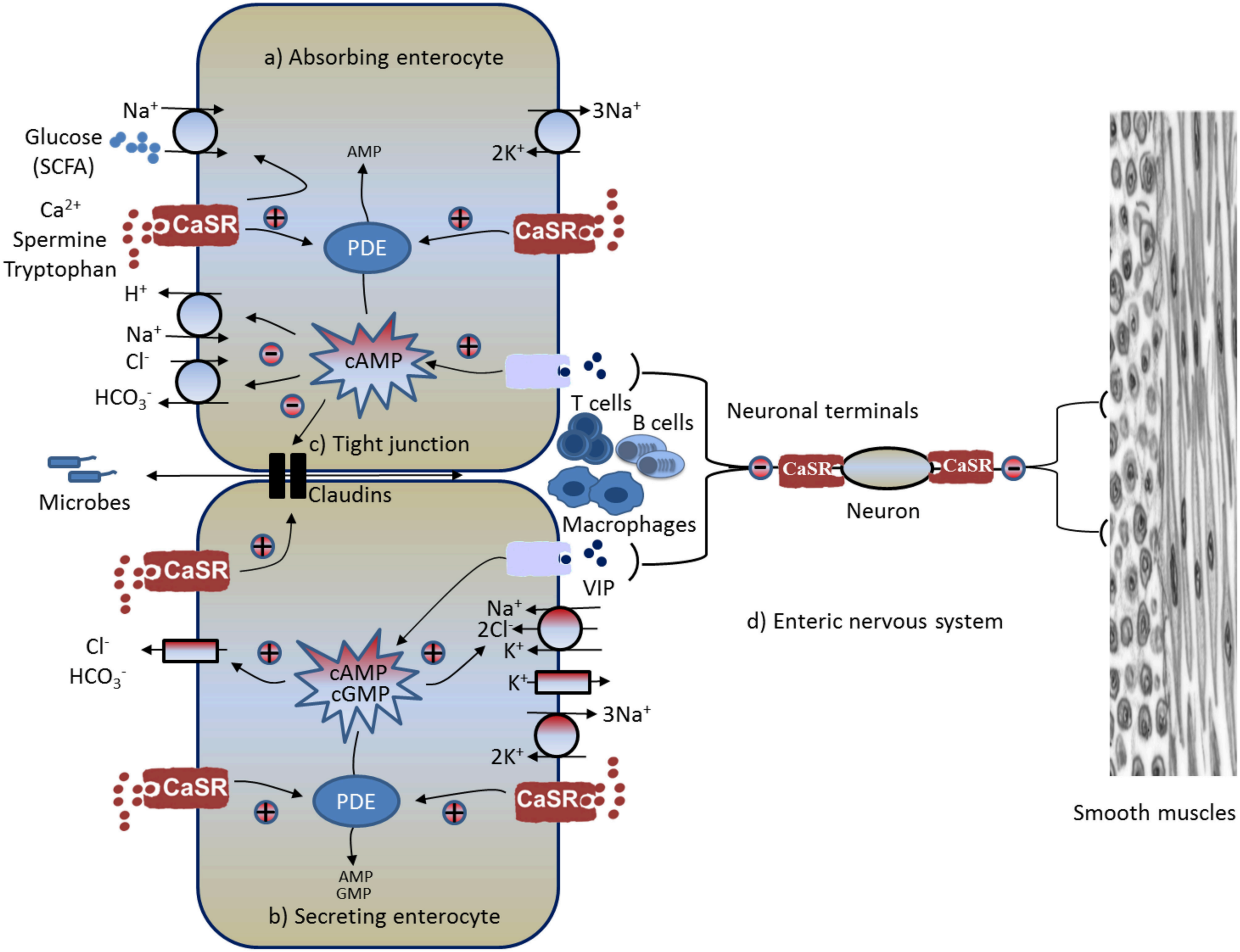
**Schematic diagram illustrating pathways and mechanisms through which CaSR-activating calcimimetics and agonists modulate GI physiology and immunophysiology**. Known CaSR effects include: **(A)** increased absorption, **(B)** decreased secretion, **(C)** enhanced intestinal barrier and reduced inflammation, and **(D)** reduced enteric nerve activity and motility (see text for explanations). CaSR, calcium-sensing receptor; CFTR, cystic fibrosis transmembrane conductance regulator; PDE, phosphodiesterase; SCFA, short-chain fatty acid; VIP, vasoactive intestinal peptide; +, stimulation; –, inhibition.

In addition to digesting food, the GI tract moves a large amount of fluid and electrolytes. Accordingly, CaSR may also regulate intestinal fluid homeostasis and electrolyte balance. Normally, fluid moves *across* and *along* the intestine. While the fluid movement *across* the intestine [absorption (Figure 1A) or secretion (Figure 1B)] is driven by active epithelium transport of electrolytes (mainly Na^+^, Cl^−^, and ${\text{HCO}}_{3}^{-}$) and solutes (mainly glucose in the small intestine and SCFAs in the large intestine), the fluid moving *along* the intestine (anterograde or retrograde) is governed by gut motility (see Figure 1D). The ENS, the brain of the gut, controls both processes, with absorption/secretion regulated by the submucosal Meissner's plexus, and the motility under the control of the myenteric Auerbach's plexus (see Figure 1D). CaSR has been identified on the apical and basolateral membranes of fluid-absorbing villous/surface cells and fluid-secreting crypt cells of rat and human intestines (Chattopadhyay et al., 1998; Cheng et al., 2002), as well as on the Meissner's and Auerbach's plexuses of the ENS (Chattopadhyay et al., 1998; Cheng, 2012). Receptors in both membrane domains of these polarized epithelia, as well as the ENS, are functionally active (Chattopadhyay et al., 1998; Cheng et al., 2002; Cheng, 2012; Tang et al., 2015) and can be activated by ${\text{Ca}}_{\text{o}}^{2+}$ (Cheng et al., 2002, 2004; Geibel et al., 2006), polyamines (Cheng et al., 2004), and the specific pharmacologic CaSR agonist R568 (a calcium mimetic drug; Geibel et al., 2006; Tang et al., 2015), suggesting its likely role in regulation of intestinal fluid metabolism.

### Activation of CaSR inhibits anion secretion

The first evidence that suggests the modulation of intestinal fluid metabolism by CaSR was made in perfused crypts isolated from rat colons (Cheng et al., 2002). In this study, the effect of increasing either luminal or basolateral ${\text{Ca}}_{\text{o}}^{2+}$ on the direction and rate of net fluid movement (^*net*^*J*_*v*_) was determined in isolated rat distal colonic crypts in both basal and forskolin (cAMP)-stimulated states. Colonic crypt is a typical fluid-transporting epithelium that is able to move fluid in two directions. Depending on the presence or absence of secretagogues and/or anti secretagogues and their relative forces in the milieus, the fluid can be transported either from the luminal side to the vascular side, resulting in net fluid absorption or positive ^*net*^*J*_*v*_, or from the blood side to the luminal side, resulting in net fluid secretion or negative ^*net*^*J*_*v*_. The study showed that, in the absence of the secretagogue, the colonic crypt exhibited a positive ^*net*^*J*_*v*_, indicating net fluid absorption; exposure of crypts to the cAMP-elevating secretagogue forskolin induced net fluid secretion; increasing ${\text{Ca}}_{\text{o}}^{2+}$ in either the luminal or vascular perfusate abolished the stimulatory effect of forskolin on net fluid secretion. In the presence of increased ${\text{Ca}}_{\text{o}}^{2+}$, net fluid secretion induced by forskolin was completely reversed, resulting in net fluid absorption (Cheng et al., 2002). Although the concentrations of ${\text{Ca}}_{\text{o}}^{2+}$ used in this study (2 and 5 mM) were slightly higher than the ${\text{Ca}}_{\text{o}}^{2+}$ concentrations in the blood and may be less physiological, the finding suggested a potential role for ${\text{Ca}}_{\text{o}}^{2+}$ acting via CaSR in regulating intestinal fluid movement.

A subsequent study used this CaSR-modulated response to further assess the interactions of spermine and ${\text{Ca}}_{\text{o}}^{2+}$ (Cheng et al., 2004). In this study, the effect of increasing luminal or basolateral polyamine on forskolin-stimulated ^*net*^*J*_*v*_ was examined in the presence of a fixed dose of ${\text{Ca}}_{\text{o}}^{2+}$. Three doses of ${\text{Ca}}_{\text{o}}^{2+}$ were tested: the threshold (0.1 mM), the near physiological (0.5 mM), and the physiological (1.0 mM). Similar to increasing ${\text{Ca}}_{\text{o}}^{2+}$, addition of the polyamine spermine to either the luminal or basolateral perfusate dose dependently reversed net fluid secretion to absorption; the extent to which spermine reduced the net fluid transport depended on the concentration of ${\text{Ca}}_{\text{o}}^{2+}$. Thus, at a threshold dose of ${\text{Ca}}_{\text{o}}^{2+}$, millimolar or sub-millimolar concentrations of spermine were required to reverse the secretory ^*net*^*J*_*v*_; on the other hand, when a physiological ${\text{Ca}}_{\text{o}}^{2+}$ was present, a much lower concentration (low micromolar) of spermine was needed to reverse the direction of forskolin-stimulated ^*net*^*J*_*v*_ (Cheng et al., 2004). The latter is the polyamine concentration most often seen in breast milk (Pollack et al., 1992; Romain et al., 1992; Buts et al., 1995) but not in infant formulas [in which the polyamine concentration is at least 1 order of magnitude lower than in breast milk and 2–3 orders of magnitude lower than the polyamine concentration in the lumen of the intestine shortly after ingestion of a typical adult human meal (see reviews Bardócz et al., 1995; Ralph et al., 1999; Milovic, 2001)]. Thus, it is conceivable that supplementation of oral rehydration solution or infant formulas with polyamines may be beneficial in treating children with diarrhea.

A definitive study that established the role for the colonic CaSR in regulating intestinal fluid secretion was the comparison of secretagogue-stimulated ^*net*^*J*_*v*_ responses to ${\text{Ca}}_{\text{o}}^{2+}$ and R568 in colonic crypts of CaSR null mice (*Casr*^−∕−^*Gcm2*^−∕−^) with the wild type controls (*Casr*^+∕+^*Gcm2*^+∕+^) (Geibel et al., 2006). It showed that CaSR, activated from either the mucosal or serosal side by ${\text{Ca}}_{\text{o}}^{2+}$ or R568, reduced secretagogue-stimulated net fluid secretion in colonic crypts of wild type mice, but not in colonic crypts from CaSR null mice. In CaSR null mice, colons responded to cholera toxin or guanylin with stimulated fluid secretion but, unlike the wild type controls, failed to generate the inhibitory actions of ${\text{Ca}}_{\text{o}}^{2+}$or R-568 (Geibel et al., 2006). Speculating that CaSR may inhibit fluid secretion through inhibiting cyclic nucleotide accumulation or metabolism, levels of colonocyte cAMP, and cGMP were measured. As expected, stimulation of adenylyl cyclase with forskolin or cholera toxin increased cytosolic cAMP, and stimulation of guanylyl cyclase with guanylin or the *Escherichia coli* heat-stable enterotoxin STa increased cytosolic cGMP; increased ${\text{Ca}}_{\text{o}}^{2+}$ or R568 abolished these effects (Geibel et al., 2006). Similar inhibitory effects were seen for spermine in isolated human colonic mucosa (Rogers et al., 2015). Interestingly, the CaSR-mediated inhibitory effects on cyclic nucleotides as well as on increased fluid secretion were prevented by the phosphodiesterase (PDE) inhibitor IBMX (Geibel et al., 2006). Similar preventative effects on CaSR were also produced by inhibition of phospholipase C (PLC) by U73122 (Geibel et al., 2006) or by depletion of inositol trisphosphate (IP_3_)-sensitive intracellular Ca^2+^ stores by thapsigargin (Cheng et al., 2002). These *in vitro* studies strongly support the notion that activation of CaSR in the colon inhibits fluid secretion through receptor-mediated destruction of cyclic nucleotides by PDE using a signaling pathway that activates PLC-IP_3_- ${\text{Ca}}_{\text{i}}^{2+}$ (see Figure 1B).

#### Cl^−^ secretion

Fluid secretion is driven primarily by transepithelial anion (e.g., Cl^−^) secretion (Barrett and Keely, 2000; Kere and Höglund, 2000; Kunzelmann and Mall, 2002). The next question that was examined was whether CaSR activation inhibits transepithelial Cl^−^ secretion. The secretion of Cl^−^ into the lumen of the intestine requires two separate, but interconnected movements: Cl^−^ entry from the blood into the cell and Cl^−^ exit from the cell into the lumen (see Figure 1B). The egress of the anion is conducted through apical membrane anion channels primarily the cAMP-dependent, 5-nitro-2-(3-phenylpropylamino) benzoic acid (NPPB)/glibenclamide-sensitive cystic fibrosis transmembrane conductance regulator chloride channels (CFTR), whereas the entry of Cl^−^ is critically dependent on the activity of the basolateral membrane bumetanide-sensitive Na^+^-K^+^-2Cl^−^ cotransporter (NKCC1; Kunzelmann and Mall, 2002). Mice deficient in CFTR lack a secretory response to cholera toxin (Gabriel et al., 1994); similarly, mice lacking NKCC1 exhibit blunted secretion to cAMP or STa (cGMP) challenge (Flagella et al., 1999). By measuring short circuit current (*I*_*sc*_) responses to pharmacological inhibitors of anion channels in the apical membrane of colonic mucosa mounted in Ussing chambers, it was shown that CaSR activation inhibited the cAMP-dependent, NPPB/glibenclamide-sensitive, apical anion channel activity (Tang et al., 2015). Likewise, by measuring Cl^−^-sensitive MQAE fluorescence responses in perfused colonic crypts, it was also demonstrated that secretagogue-induced, bumetanide-sensitive, basolateral membrane Cl^−^ entry into colonocytes was inhibited by either R568 or by increasing [${\text{Ca}}_{\text{o}}^{2+}$] of the basolateral bath fluid, consistent with CaSR inhibition of NKCC1 (Geibel et al., 2006). Therefore, in colons CaSR inhibits both Cl^−^ entry and exit. Currently, it remains unknown whether CaSR directly inhibits these ion transporters or indirectly via the reversal of changes in cyclic nucleotide by the activation of the receptor.

#### ${\text{HCO}}_{3}^{-}$ secretion

In addition to Cl^−^ secretion, ${\text{HCO}}_{3}^{-}$ secretion is increased upon secretagogue stimulation, and this stimulated ${\text{HCO}}_{3}^{-}$ secretion contributes to alkaline deficit and metabolic acidosis, as well as to systemic volume depletion and dehydration seen in cholera and other diarrhea conditions (Fordtran, 1967; Powell et al., 1971). Regulated ${\text{HCO}}_{3}^{-}$ secretion is also essential for mucosal defense against luminal acid (via neutralization) in the upper GI tract and bacteria (via stimulation of mucus secretion and maintenance of intestinal barrier function) in the lower GI tract; defects in ${\text{HCO}}_{3}^{-}$ secretion have been shown to be a risk factor for peptic ulcer diseases (Isenberg et al., 1987; Flemstrom and Isenberg, 2001; Allen and Flemström, 2005) and intestinal inflammation (Garcia et al., 2009; Xiao et al., 2012, 2013). To assess if CaSR activation inhibits secretagogue-induced ${\text{HCO}}_{3}^{-}$ secretion, colonic mucosa ${\text{HCO}}_{3}^{-}$ secretory response to R568 was also studied recently (Tang et al., 2015). In this study, the cAMP-elevating secretagogue forskolin was employed to induce ${\text{HCO}}_{3}^{-}$ secretion, and ${\text{HCO}}_{3}^{-}$ secretion was monitored both electrophysiologically by recording ${\text{HCO}}_{3}^{-}$ ion-dependent short-circuit current (*I*_*sc*_) and chemically through measuring the rate of ${\text{HCO}}_{3}^{-}$ secretion (*J*_*HCO*3_) using a so-called “pH stat” technique. The latter measures the amount of exogenous acid (HCl or H_2_SO_4_) delivered per hour per cm^2^ surface area to neutralize the secreted alkaline (${\text{HCO}}_{3}^{-}$) in order to maintain a constant lumen fluid pH. It was found that forskolin stimulated both *I*_*sc*_ and *J*_*HCO*3_ in colonic mucosa of rats, wild type mice, and mice lacking the intestinal epithelium CaSR (^villin^*Cre/Casr*^flox∕flox^; Tang et al., 2015), consistent with active control of ${\text{HCO}}_{3}^{-}$ secretion in the intestine by cyclic nucleotide. However, subsequent activation of CaSR, either apically or basolaterally by R568, significantly reduced forskolin-induced ${\text{HCO}}_{3}^{-}$ secretion in colonic mucosa of rats and wild type mice, but not ^villin^*Cre/Casr*^flox∕flox^ (Tang et al., 2015), suggesting the involvement of CaSR in the inhibition of ${\text{HCO}}_{3}^{-}$ secretion. These studies established that CaSR regulates intestinal ${\text{HCO}}_{3}^{-}$ secretion.

### Activation of CaSR enhances salt and solute absorption

CaSR is also expressed in absorbing villus/surface cells (Table 1), suggesting critical roles in regulating intestinal absorption (see Figure 1A). To address this, colonic crypts or mucosa from rat and mice were isolated, and effects of CaSR agonists on absorption were examined (Geibel et al., 2006). Bidirectional fluid movements (absorption and secretion) take place in the same enterocytes. To minimize interference from secretion, in these studies tissues were first treated with basolateral bumetanide to block secretion before absorption was investigated. Without secretagogues, perfused crypts exhibited a positive net *J*_*v*_ (^*net*^*J*_*v*_), suggesting that under baseline conditions, colonic crypts are in a net fluid absorption mode. Addition of bumetanide to the basolateral bath fluid of perfused crypts slightly increased the absorptive ^*net*^*J*_*v*_, consistent with inhibition of a small component of fluid secretion that remains under basal non-stimulated condition. This low basal fluid secretion is likely attributable to the low cyclic nucleotide level that remains even in the absence of secretagogues. Thus, in the presence of bumetanide, ^*net*^*J*_*v*_ measurements represent the absorptive component of fluid transport. This absorptive fluid movement was substantially reduced by exposure to cAMP or forskolin. Activation of CaSR by either increasing ${\text{Ca}}_{\text{o}}^{2+}$ and/or addition of R568 to the basolateral bath significantly reduced the cAMP-mediated reduction in absorptive ^*net*^*J*_*v*_, demonstrating that activation of CaSR not only suppresses the stimulated fluid secretion but also reverses the reduced fluid absorption.

#### Na^+^ absorption

Solute absorption in the colon (and the small intestine) is driven primarily by parallel Na^+^/H^+^ (sodium-hydrogen exchanger, NHE) and Cl^−^/${\text{HCO}}_{3}^{-}$ exchange located at the apical plasma membranes (see Figure 1A). It is known that this Na^+^-dependent fluid absorption in the colon, as well as in the ileum, is reduced by cholera toxin and other cyclic nucleotide-elevating enterotoxins via inhibiting NHE activity and Cl^−^/${\text{HCO}}_{3}^{-}$ exchange (Thiagarajah et al., 2015). It is also acknowledged that this event contributes to the severity of fluid and electrolyte losses in secretory diarrheas (Kunzelmann and Mall, 2002; Field, 2003; Thiagarajah et al., 2015). Similar to the events in secretory diarrheas, during normal digestion, vasoactive intestinal peptide (VIP) and other cyclic nucleotide-stimulating paracrines/autocrines/neurocrines are generated from enteroendocrine cells and/or the subepithelial ENS. These secretagogues stimulate intestinal secretion in distinct ion transport processes that not only increase the secretory component but also reduce the absorptive component of transepithelial fluid movement, leading to net digestive secretion (Barrett and Keely, 2000; Kunzelmann and Mall, 2002). This secretion helps to mix up food components and to lubricate the lumen surface of the intestine. To evaluate whether CaSR also modulates the NHE-mediated Na^+^ absorptive process, isolated colonocytes were preloaded with BCECF (a fluorescent probe for H^+^ or pH), and effects of CaSR agonists on Na^+^-dependent proton extrusion from the apical membrane of colonocytes (a standard measure of NHE-mediated Na^+^ absorptive activity) were studied (Geibel et al., 2006). Raising the basolateral bath ${\text{Ca}}_{\text{o}}^{2+}$ to activate CaSR significantly increased the Na^+^-dependent acid extrusion rate; the addition of R568 resulted in further elevation of this rate, demonstrating that CaSR stimulates Na^+^ absorption mediated by NHE.

#### Cl^−^ absorption

Does CaSR stimulate Cl^−^ absorption mediated by parallel Cl^−^/${\text{HCO}}_{3}^{-}$ exchange so as to match the receptor stimulation of Na^+^ absorption by the receptor (see Figure 1A)? To answer this question, in another study, transepithelial Cl^−^ absorption was measured (Tang et al., 2015). Colonic mucosa were isolated, mounted into Ussing chambers and perfused, and lumen Cl^−^-dependent ${\text{HCO}}_{3}^{-}$ secretion (a measure of Cl^−^/${\text{HCO}}_{3}^{-}$ exchange-mediated Cl^−^ absorptive activity) was recorded and compared in the presence and absence of R568 stimulation using the Ussing chamber-pH stat technique (Tang et al., 2015). Similar to the R568 effects on Na^+^/H^+^ exchange, lumen Cl^−^-dependent ${\text{HCO}}_{3}^{-}$ secretion was found to be significantly stimulated by R568 in the colons of both rats and wild type mice, but not in *CaSR*^−∕−^ mice (Tang et al., 2015).

#### SCFA absorption

Short-chain fatty acids (SCFAs) are the major anions in stool. SCFAs are produced in the colon by bacterial fermentation of unabsorbed carbohydrates. SCFA absorption stimulates Na^+^, Cl^−^, and water absorption and represents a major mechanism in the colon to conserve fluid and electrolytes (see Figure 1A). SCFA absorption occurs via a process involving apical membrane Na^+^/H^+^, Cl^−^/${\text{HCO}}_{3}^{-}$, and SCFA/${\text{HCO}}_{3}^{-}$ exchanges (Ruppin et al., 1980; Binder, 2010), and Na^+^/H^+^ and Cl^−^/${\text{HCO}}_{3}^{-}$ exchanges are stimulated by activation of CaSR. This combined knowledge led to a hypothesis that CaSR activation also stimulates SCFA/${\text{HCO}}_{3}^{-}$ exchange and enhances SCFA absorption. To examine this, in a separate experiment using the same aforementioned Ussing chamber—pH stat technique, SCFA absorption mediated by SCFA/${\text{HCO}}_{3}^{-}$ exchange was measured as lumen isobutyrate-dependent ${\text{HCO}}_{3}^{-}$ secretion (Tang et al., 2015). Comparable to the R568 effects on Na^+^/H^+^ and Cl^−^/${\text{HCO}}_{3}^{-}$ exchanges, isobutyrate-dependent ${\text{HCO}}_{3}^{-}$ secretion was also found to be significantly stimulated by R568 in the colons of rats and wild type mice, but once again not *CaSR*^−∕−^ mice (Tang et al., 2015).

Jointly, these studies have established that CaSR is a regulator of NaCl and SCFA absorption in the colon. These findings are not surprising considering that CaSR has been shown to be used by chloride cells of gills, as well as by ion transporting cells of the kidney and the gut of marine and freshwater fish alike in order to pump Cl^−^ and other ions to direct water flow (Nearing et al., 2002); however, these new data provide compelling support for the notion that CaSR is an important regulator of intestinal fluid movement. In order to generate the large quantities of fluid needed for the digestion of ingested food, the gut has evolved a complex series of neuronal, hormonal, and/or paracrine/autocrine feedback regulation mechanisms that allow for the continued production of fluid during the phases of ingestion and digestion. Moreover, when digestion is complete, the gut signals for the induced secretion to stop and for subsequent absorption to occur. The studies presented suggest that the nutrients released from food as a result of digestion may act as signals that activate CaSR in the gut epithelium, and probably also CaSR in the ENS (see below), thereby initiating and coordinating transition of these processes.

Besides the aforementioned transporters, apical Na^+^ and K^+^ channels, as well as basolateral K^+^ channels and Na^+^,K^+^-ATPase (Figure 1), also play critical roles in epithelial absorption and secretion. For example, in Cl^−^ secreting epithelia, the basolateral K^+^ channels facilitate basolateral Cl^−^ entry (via cycling back the K^+^ for NKCC1), as well as apical Cl^−^ exit (by maintaining a favorable transepithelial electrical gradient), while Na^+^,K^+^-ATPase pumps the Na^+^ entered by NKCC1 out of the cell. Whether CaSR also affects the activity and function of these transporters remains to be determined.

Also needed to be studied are CaSR effects *in vivo*. Most of the studies so far are based on observations from perfused crypts or use of Ussing chambers. Studying these effects in isolation can be artificial. Future studies should be directed at the organismal and systemic levels in order to better define the net effect of the absence of the gene. In this respect, studies that characterize the phenotype of CaSR^−∕−^ mice (Rey O et al., 2012; Macleod, 2013b; Cheng et al., 2014) or associate SNPs in CaSR for diarrheal conditions (Ho et al., 2010; Romero et al., 2015) would be useful. Nonetheless, preliminary data from animals (Bovee-Oudenhoven et al., 1996, 1997; Bovee Oudenhoven et al., 1997, 1999) and clinical trials on humans (Bovee-Oudenhoven et al., 2003; Cheng et al., 2013; Dadu et al., 2015) using calcium or other CaSR ligands indicate that activation of this nutrient sensing receptor *in vivo* does reduce symptoms of infectious diarrheas (also see recent review by Cheng, 2016).

With regards to the mechanisms for the effects of CaSR on ion transport, it has been postulated that CaSR agonists enhance cyclic nucleotide destruction via G protein-mediated activation of PDE, instead of inactivation of adenylyl cyclase found in non-intestinal cells (Geibel et al., 2006). Consistent with this notion, inhibition of net fluid secretion by CaSR agonists correlated with reductions in cyclic nucleotide accumulations, both of which were abolished by IBMX (Geibel et al., 2006). IBMX is a non-specific PDE inhibitor. It remains unclear which specific isoform of PDE in the intestine mediates the CaSR action. Also unknown is the mechanism by which CaSR activates PDE. There are 11 families of cyclic nucleotide-degrading PDEs, each exhibiting selective affinities for degrading cAMP and/or cGMP (Beavo, 1995; Jeon et al., 2005). Although little information is available on the specific PDE genes expressed in intestinal epithelial cells, PDE1-5 isoforms have been identified in human colon cancer cells (Soh et al., 2000; O'Grady et al., 2002), and each of these PDEs is sensitive to IBMX. Because inhibition of PLC signaling abolishes CaSR effects on cAMP and cGMP accumulation (Geibel et al., 2006), it is tempting to speculate that activation of ${\text{Ca}}_{\text{i}}^{2+}$/calmodulin-dependent PDE1 may be responsible. Future work is needed to determine whether PDE1 is activated by CaSR, and if so, how it is activated. Also needed to be elucidated are the mechanisms for how CaSR regulates and coordinates distinct ion channels and transporters in secretion and absorption, and whether CaSR regulates absorption by surface/villous cells using the same or different mechanism than CaSR regulates secretion by crypts. CaSR binds a plethora of ligands, interacts with multiple G protein subtypes, and regulates divergent downstream signaling pathways. It is possible that CaSR may use a completely different mechanism to regulate the same type of function in a different cellular context. For example, a recent study by Xie et al. (2014) showed that in the duodenum CaSR seems to employ an alternative intracellular signaling pathway to increase ${\text{HCO}}_{3}^{-}$ secretion in this tissue. This alternative pathway involves ${\text{Ca}}_{\text{i}}^{2+}$-dependent activation of receptor-operated channel, intermediate conductance K^+^ channels, and CFTR. Similarly, Brennan et al. (2016) recently reported CaSR stimulation of CFTR in developing human fetal lung. This stimulatory effect of CaSR appears to be mediated through a ${\text{Ca}}_{\text{i}}^{2+}$-dependent adenylyl cyclase.

## CaSR and ENS activity and motility

As previously mentioned, the ENS is the brain of the gut. The ENS comprises two plexuses—the submucosal Meissner's plexus and the myenteric Auerbach's plexus. While the former controls secretion, the latter regulates motility (see Figure 1D). CaSR is expressed in both plexuses (nerve fibers and somas; Chattopadhyay et al., 1998; Cheng et al., 1999; Cheng, 2012), suggesting its potential role in controlling the activity and functions of these enteric neurons.

### CaSR inhibits ENS activity and ENS-mediated secretion

In mammals (humans and rodents), as much as 80–90% of the fluid secreted at basal condition, during digestion, and in diarrheas (cholera and rotavirus) are ENS-mediated (Burleigh and Borman, 1997; Lundgren et al., 2000; Lundgren, 2002; Field, 2003; Farthing, 2006; Lorrot and Vasseur, 2007). These estimates are based on the magnitude to which the fluid secreted or current evoked is inhibited by tetrodotoxin (TTX) or lidocaine. As selective inhibitors of voltage-gated Na^+^ channels in neural tissues, TTX and lidocaine are used to inhibit neurotransmission, and subsequently ENS activity. Using this method, TTX-sensitive *I*_*sc*_ responses in freshly isolated, intact ENS-containing rat colon segments mounted onto Ussing chambers were measured, and effects in the presence versus absence of serosally added R568 were compared (Cheng, 2012; Cheng et al., 2012). In these experiments, TTX-sensitive *I*_*sc*_ was employed as a measure of ENS activity and ENS-mediated anion secretion, and serosal R568 was applied to activate CaSR in the ENS. Consistent with active regulation of secretion by the ENS, a large portion (~70–75%) of *I*_*sc*_ in the proximal and distal colon was inhibited by TTX, both at basal and under cAMP (forskolin or cholera toxin)-stimulated conditions. Furthermore, the addition of forskolin or cholera toxin increased TTX-sensitive *I*_*sc*_; subsequent addition of R568 to the serosal bath to activate CaSR in the ENS abolished the secretagogue-stimulated TTX-sensitive *I*_*sc*_. Similar effects of R568 addition were also observed on basal TTX-sensitive *I*_*sc*_. These results demonstrate that CaSR agonists function as inhibitors of ENS activity, reducing ENS-mediated secretion. These findings also point to a dual model for regulating intestinal fluid transport, in which neuronal and non-neuronal secretagogue actions are modulated by the inhibitory effects of CaSR on the ENS—which is TTX/lidocaine-sensitive—as well as by CaSR on the epithelium—which is TTX/lidocaine-insensitive. Clearly, further studies are needed to address these possibilities. Also, future experiments will be required to understand which type of neuron(s) in the ENS expresses and is affected by CaSR and to determine what the intracellular second messenger mechanism(s) is involved in this process.

### CaSR inhibits motility

CaSR is also present in the myenteric Auerbach's plexus that governs gut motility (Chattopadhyay et al., 1998; Cheng, 2012). Thus, CaSR may play a role in regulating motility as well. In support of this notion, people who take high calcium are often constipated (Prince et al., 2006), as are patients with hypercalcemia (Ragno et al., 2012). Likewise, animals receiving treatment of polyamines (e.g., spermine) or peptides (e.g., γ-glutamyl cysteine), both of which are classes of agonists for CaSR, display profound inhibition on gastric emptying and/or intestinal motor activity (Belair et al., 1981; Tansy et al., 1982; Muramatsu et al., 2014). Polyamine inhibition of GI transit is also noted in several rodent models of diarrhea-dominate forms of irritable bowel syndrome (Bergeron et al., 1996, 2001a,b). Together, these studies highlight the importance of this nutrient sensing receptor in modulating ENS activity and gut motility.

The physiological and pathophysiological significance of these findings remains to be determined. Since the enteric nerve network follows the nutrient-carrying blood and lymph vessel networks, CaSR may provide a mechanism for the ENS to sense nutrients and fine-tune its activity and function in accordance with the status of digestion and absorption. For example, upon food ingestion, mechanical signals activate the ENS, enhancing its functions in promoting secretion and motility to facilitate digestion; upon completion of digestion, the chemical signals (e.g., absorbed nutrients) carried in the parallel blood or lymph vessels may activate CaSR to deactivate the ENS, thereby slowing down secretion and motility in order to facilitate absorption. Considering its normal role in the gut, CaSR may represent an excellent candidate gene to be investigated in the pathophysiology of irritable bowel syndrome (IBS), a common clinical condition characterized by chronic diarrhea and/or constipation, in addition to abdominal pain and cramping. Given the key CaSR function in regulation and coordination of secretion and muscular contraction, it is possible that over activity of the CaSR may result in under-activation of the ENS, leading to the constipation-dominate form of IBS (IBS-C). Likewise, reduced activity of the CaSR may result in excessive activation of the ENS, leading to the diarrhea-dominate form of IBS (IBS-D). Clearly, more work is needed to verify these hypotheses in IBS patients, like the one that has recently been published (Romero et al., 2015) even if this latter study revealed no association between the common CaSR polymorphism rs1801725 and IBS.

## CaSR and intestinal barrier integrity and immunity

In addition to modulating the absorption of nutrients and secretion of electrolytes and fluids, the intestinal epithelial barrier, with its intercellular tight junctions, also controls the passage of gut microbes across the mucosa. This is to avoid over-activating the sub-epithelial immune cells, mainly dendritic cells (DCs), T cells, and B cells, thereby preventing local and systemic inflammation (Turner, 2006). More recently, our laboratory illustrated the role of CaSR and the consequence of its manipulation both locally and systemically using intestinal epithelium-specific *CaSR*^−∕−^ mice (^villin^*Cre/Casr*^flox∕flox^; Cheng et al., 2014). We found that epithelial CaSR is also an important regulator of intestinal barrier integrity and immunity, as well as a key modulator of gut bacteria-sensing. Epithelial CaSR deficiency resulted in diminished intestinal barrier function, altered composition and distribution of the gut bacteria, and skewed immunity toward a pro-inflammatory response (Cheng et al., 2014). These observations strongly support the notion that the epithelial barrier plays an important role in triggering immune activation and gut inflammation.

### CaSR contributes to intestinal barrier integrity

In contrast to transcellular transport, in which ions and solutes travel *through* the epithelial cell passing through both the apical and basolateral membranes, paracellular transport refers to the transfer of ions and substances across the intestinal epithelium by passing through the intercellular space *between* the cells. Situated between adjacent intestinal epithelial cells of the mucosa are structures called apical junctional complexes, such as tight junctions (TJs; Figure 1C). These intercellular structures, along with the layer of epithelium composing the intestinal mucosa, act as a barrier separating the luminal contents from the submucosal compartment, which is home to the gut immune system. TJs harbor complex interactions between ~40 proteins, including the transmembrane proteins occludins and claudins. These proteins are anchored to the actin filaments and myosin light-chain through the zonula occludens family. Breaching of this dynamic barrier may result in excessive exposure of the gut immune system to luminal microbes and foreign antigens, leading to intestinal inflammation (Turner, 2006). Since it is well-known that ${\text{Ca}}_{\text{o}}^{2+}$ is required for the development (Martinez-Palomo et al., 1980; Cereijido et al., 1981; Gonzalez-Mariscal et al., 1985) and the maintenance (Galli et al., 1976; Meldolesi et al., 1978; Palant et al., 1983) of stable epithelial TJs between epithelial cells, we hypothesized that CaSR may participate in regulating TJ assembly/formation and paracellular permeability.

**Figure 2:**
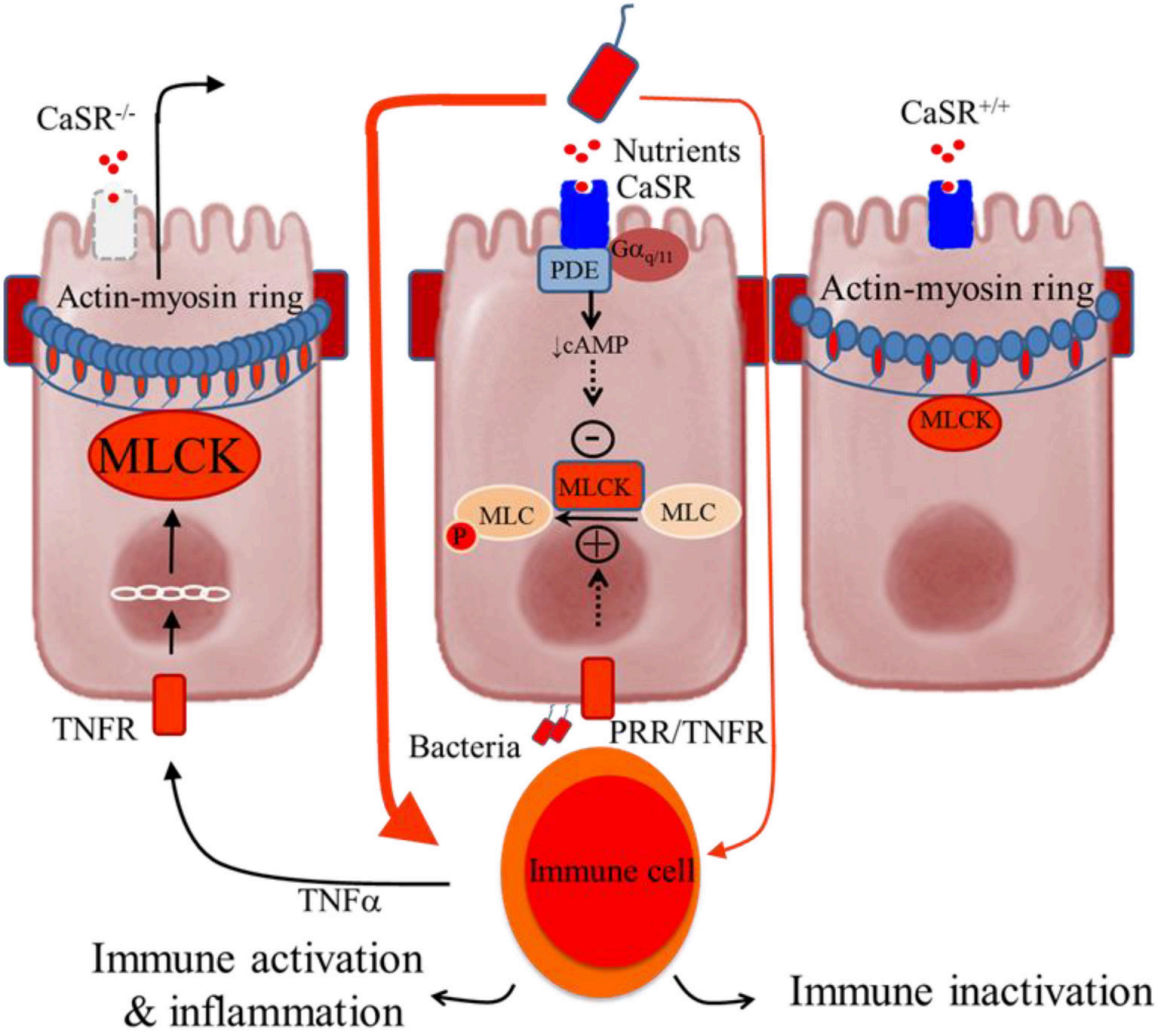
**Schematic representation of the colonocytes showing how deficiency in intestinal CaSR results in increased gut permeability and inflammation**. **Central panel**, illustrates a current model of self-amplifying pathway for intestinal disease (Turner, 2006) where a small amount of luminal bacteria or bacteria-derived molecules pass across the epithelium to activate lamina propria immune cells, leading to secretion of proinflammatory cytokines (e.g., TNFα) and subsequent activation of their receptors (e.g., TNFR) in the epithelium. The latter increases MLCK transcription and activity and phosphorylation of myosin light chain (MLC), resulting in increased contractility of perijunctional actin-myosin ring and increased epithelial permeability. The consequences are greater access leakage of luminal materials, greater immune activation, and even greater barrier defects. The presence of CaSR ligands and signals limits amplifying of this cascade through activation of phosphodiesterase (PDE) (Geibel et al., 2006) and inhibition of MLCK (Cheng et al., 2014), leading to MLC dephosphorylation and barrier stabilization. Thus, in the presence of intact CaSR signaling, as in CaSR^+∕+^ mice, immune tolerance or only low-grade inflammation is seen **(Right panel)**. However, in the absence of CaSR signal, as in CaSR^−∕−^ mice, the limiting of this cascade amplification is lost, leading to immune activation and uncontrolled inflammation (Macleod, 2013b; Cheng et al., 2014) **(Left panel)**.

Figure 2 shows the hypothesized mechanism for how CaSR produces its effects on the control of intestinal epithelial barrier permeability and inflammation. According to a current model of IBD (Turner, 2006), intestinal inflammation is induced by a self-amplifying pathway where a limited amount of luminal bacteria or bacteria-derived molecules cross the epithelium to activate lamina propria immune cells. This leads to secretion of pro-inflammatory cytokines (e.g., TNFa) and subsequent activation of their receptors (e.g., TNFR) in the epithelium. The latter increases myosin light-chain kinase (MLCK) transcription via the IKKβ, IκBα, and NF-κB signalosome, and then phosphorylates the regulatory myosin light chain (MLC). The result of this is increased contraction of a band of actin and myosin (actin-myosin ring) located at the tight junctions, which subsequently promotes epithelial permeability. The consequences include greater access leakage of luminal materials, greater immune activation, and even greater barrier defects. CaSR limits the amplification of this cascade through receptor-mediated Gα_q∕11_-dependent activation of PDE (Geibel et al., 2006) and inhibition of NF-κB phosphorylation. As a result, MLCK is down-regulated, MLC phosphorylation inhibited, and epithelial barrier stabilized. Hence, in the presence of intact CaSR signaling, immune inactivation or tolerance is maintained. Conversely, in the absence of CaSR, ligands, or signals, as in *CaSR*^−∕−^ mice, this cascade is self-amplified, leading to immune activation and uncontrolled inflammation.

To test this hypothesis, we examined the intestinal permeability of *CaSR* null mice (^villin^*Cre/Casr*^flox∕flox^) lacking CaSR in the intestinal epithelium *ex vivo* using the aforementioned Ussing chamber technique (Cheng et al., 2014). Compared to wild type controls, *CaSR*^−∕−^ mice displayed significantly lower transepithelial electrical resistance, higher conductance, and higher passive transport of FITC-conjugated dextran (Cheng et al., 2014). Surprisingly, significant alterations in *I*_*sc*_ and ion transporter transcript expressions, indicative of defective transcellular transport, were not observed in *CaSR*^−∕−^ mice (Cheng et al., 2014). This suggests that, unless in minute-to-minute regulations—as detailed in previous sections—in the setting of long-term regulation, CaSR appears to affect the paracellular transport pathway exclusively.

Consistent with a defective intestinal barrier, *CaSR*^−∕−^ mice showed decreased colonic epithelial expression of TJ molecules, particularly *claudin-2*, a major component of TJs, whereas expression of MLCK-1 was found to be significantly increased (Cheng et al., 2014). Also increased was the expression of the TJ protein *Cingulin* in *CaSR*^−∕−^ mice compared to their wild type counterparts (Cheng et al., 2014). Although the exact function is unknown, *Cingulin* has been associated with the expression of occludin and claudins, and the epithelial specific transcription factors *Gata4, Gata6* and *Hif-1*α, which is a feature also embodied by *CaSR*^−∕−^ mice (Cheng et al., 2014).

### CaSR regulates gut bacteria sensing and balance

Besides handling dietary nutrients, the mammalian gut harbors a huge number (10^13^–10^14^) of microorganisms, collectively known as microbiota, especially in the colon. With the utilization of improved techniques measuring gut microbiota composition and function, such as 16S rDNA high-throughput sequencing, a growing number of research studies have shed light on the mutual relationship and bidirectional interactions between gut microbiota and nutrition (see recent reviews Flint et al., 2012; Maukonen and Saarela, 2015). For example, while energy extraction and metabolism are greatly influenced by these gut microbes, the composition and activity of these microbes are also substantially affected by the energy (diet and nutrients) one consumes. Multiple mechanisms are proposed to explain these interactions. We hypothesized that CaSR as a sensor for many nutrients may be involved in gut bacteria-sensing and ecosystem-balancing, regulating the nutrition-microbial-host interactions. To examine this, we analyzed and compared the microbiota of steady-state *CaSR*^−∕−^ and wild type mice using the 16S rDNA sequencing technique. We found that deficiency in epithelial CaSR altered the gut's microbe balance (Cheng et al., 2014). At the phylum level, an outgrowth in Deferribacteraceae was noted, which has previously been found to correlate with inflammatory responses in the colons of *Citrobacter rodentium*-infected mice (Hoffmann et al., 2009), a model of bacterial colitis. Concurrently, beneficial Lactobacilli and Clostridium were decreased in *CaSR*^−∕−^ mice (Cheng et al., 2014). Furthermore, the relative abundance and distribution of the gram-positive organism *Clostridium coccoides* was also significantly altered in *CaSR*^−∕−^ mice, with depletion noted in the lumen and enrichment in the sub-epithelial layer. Consistent with enhanced bacterial translocation and dissemination in host tissues, *CaSR*^−∕−^ mice had significantly decreased epithelial expression of *Reg3*β and *Reg3*γ, which encode secreted C-type lectins that bind and protect against translocation and dissemination of Gram-negative (van Ampting et al., 2012) and Gram-positive bacteria (Cash et al., 2006; Brandl et al., 2007), respectively.

In addition to detecting “health signals” (i.e., nutrients), CaSR has recently been shown to be involved in the detection of “danger signals” derived endogenously [e.g., Ca^2+^ released as a result of tissue injury (Lee et al., 2012)] or exogenously [e.g., chitin and chitosan (Huang et al., 2015; Muanprasat et al., 2015)]. Since these endogenous and exogenous danger signals are two types of molecules that belong to so-called damage-associated molecule patterns (DAMPs) and pathogen-associated molecule patterns (PAMPs), and are also ligands of the primitive pattern recognition receptors (PRRs), it is reasonable to speculate that CaSR and PRR—two ancient basic sensing mechanisms—may communicate in a way to detect an appropriate level of danger in the gut. Indeed, intestinal epithelial cells were found to express all PRRs that recognize different ligands. These include Toll-like receptors (TLRs), C-type lectins, nucleotide-binding domain and leucine-rich repeat-containing receptors (NLRs), and retinoic acid-inducible gene I-like receptors (RLRs) (Abraham and Medzhitov, 2011; Cheng et al., 2014). When CaSR function was lost, all the PRRs were up-regulated (Cheng et al., 2014). Thus, it is likely that CaSR may normally antagonize PRR signaling and keep the latter in check. Alternatively, PRRs may be up-regulated in *CaSR*^−∕−^ mice to compensate for the lost CaSR function. Similar studies that examine CaSR expression and function in PRR-deficient mice would help to address these possibilities.

Of the greatest relevance is probably the ability of the microbiota to produce polyamines, spermine (tetra-amine), spermidine (tri-amine), and their di-amine precursor putrescine. As alkaline molecules, these polycationic polyamines are fully protonated in physiologic solutions so that they resemble multivalent metal cations Ca^2+^ and Mg^2+^ and can thereby participate in many biological processes required for the maintenance of gut epithelium health (Dufour et al., 1988; Buts et al., 1993; Loser et al., 1999). In the gut, while virtually every microbiome has genes for polyamine synthesis, the major polyamine-producers are Bacteroides, Enterobacteria, and Fusobacterium (Noack et al., 1998; Sabater-Molina et al., 2011). Among the three, Bacteroides is the most predominant species of the gut flora, particularly in those individuals who consume protein and animal fat. Pectin, fructans, and other indigestible carbohydrates and dietary fiber (collectively called prebiotics) are known to be beneficial to the host. It has been postulated that this benefit is attributed to their fermentation end products short-chain fatty acids. New emerging evidence, however, suggests that prebiotics are also beneficial because they select polyamine-producing microflora (Noack et al., 1998; Delzenne et al., 2000; Sabater-Molina et al., 2011). In the colon, polyamines, specifically spermine, are potent positive allosteric modulators of CaSR (Cheng et al., 2004). In the presence of physiological or near physiological concentrations of ${\text{Ca}}_{\text{o}}^{2+}$, as low as a few nM of spermine added luminally or basolaterally was found to produce significant biological effects such as inhibition of cAMP-dependent fluid secretion by colonic crypts (Cheng et al., 2004). Thus, the bacterial polyamine-activated CaSR signaling may very well be involved in the cross-talk between the microbiome and the epithelium—an absolutely essential process required for the proper development of both innate and adaptive immune responses.

### CaSR regulates intestinal innate and adaptive immune responses

The colon is in a constant state of inflammation, the magnitude of which is controlled largely by the integrity of the intestinal barrier and the microbiota. Speculating that dysbiosis may lead to pathogenic inflammatory immune responses locally, gene array analyses of the distal colon of wild type and *CaSR*^−∕−^ mice were performed (Cheng et al., 2014). As expected, the colon of *CaSR*^−∕−^ mice showed a marked increase in expression of an array of cytokine-encoding genes, including IL-1β, TNF-α, INF-γ, IL-6, IL-12, IL-17, IL-22, IL-23, NO synthase 2, and prostaglandin E synthase 3. These changes in cytokine expression are likely attributed to the CaSR null epithelium because similar changes in expression were noted *in vitro* in cultured colonic epithelial cells as well (Mine and Zhang, 2015; Zhang et al., 2015). Increased IL-1R expression was also seen in colonic DCs in *CaSR*^−∕−^ mice, as well as in colonic CD4^+^ and CD8^+^ T lymphocytes. As further evidence of chronic intestinal inflammation in *CaSR*^−∕−^ mice, higher IL-1R and programmed cell death protein 1 (PD-1) were significantly expressed in colonic CD4^+^ and CD8^+^ T cells, and increased number of B cells with higher levels of IgA expression was found to accumulate in the colon. Moreover, compared to their wild type littermate counterparts, *CaSR*^−∕−^ mice demonstrated more severe colitis in response to challenge by dextran sodium sulfate (DSS). Their recovery was also significantly delayed, both clinically, as assessed by changes in body weight, stool consistency and stool blood, and histologically, as demonstrated by alterations in colonic inflammation and mucosal damage.

Thus, our *CaSR*^−∕−^ mice studies demonstrated that a healthy epithelial CaSR signal is required for the maintenance of a functional epithelial barrier, a symbiotic microbial-host interaction, and a balanced immune system; deficiency in CaSR results in compromised barrier function and increased translocation of bacteria that trigger the immune system, thereby potentiating this cycle, ultimately leading to inflammation.

Still, many questions remain to be addressed. For example, CaSR is lost in colon cancer (Sheinin et al., 2000; Fetahu et al., 2014, 2016). What happens to CaSR in the colons of IBD patients and animal models of DSS or infectious diarrhea? Is the expression or function of the CaSR altered as a result of inflammation or due to responsiveness to nutrients or allosteric modulators, as recently demonstrated in transformed colonic cells (Fetahu et al., 2014)? In IBD, there are significant changes to the ENS and neuroplasticity (Lomax et al., 2005); is CaSR involved? If so, what are the consequences to motility, neurosecretion, epithelial, and immune function?

## Summary and conclusions

In summary, we have shown that gut epithelium CaSR, a fundamental mechanism for sensing and regulating ionic and nutrient compositions of extracellular milieu in the small and large intestine, controls digestion, absorption, and secretion. Consequently, during the digestion phase (i.e., when food is ingested into the stomach before nutrients are released), CaSR stimulates secretion to aid in food breakdown; in contrast, during the absorptive phase (i.e., when nutritional signals are fully extracted and have reached the absorptive gut of the small intestine), CaSR stimulates absorption and inhibits secretion so as to terminate digestion and complete the cycle. We have also provided evidence that this same receptor controls intestinal permeability and immunity. Thus, whereby disrupting or inhibiting this nutrient-sensing mechanism may be associated with over-activation of the immune system and loss of immune tolerance, activating CaSR expression and activity would be helpful, not only in the maintenance of a balanced immune system, but also in deactivating the over-activated immune responses and restoring immune homeostasis. These results suggest a new paradigm for regulation of intestinal physiology and immunology, in which both fluid metabolism and immune balance may be fine-tuned by CaSR in accordance with nutrient availability and the state of digestion and absorption. Most of the studies so far are conducted *in vitro* or based on observations made from *ex vivo* tissues. Future studies need to define CaSR effects *in vivo* in animal models and transgenic mice before CaSR agonist clinical trials are performed in diarrheal patients.

Acute infectious diarrhea remains the number one cause of death among young children, particularly in developing nations. It is estimated that 1.3 million children die each year, not due to infection *per se*, but as a result of the associated diarrhea and dehydration. Although oral rehydration solution is valuable for correcting dehydration, so far there is no cost-effective therapy to stop this ongoing loss of fluid and to reduce the duration of diarrhea. Likewise, on the other end of the spectrum, chronic inflammatory, and neurogenic diarrhea, such as those caused by IBD and IBS, is a major health problem in developed countries. It is especially prevalent among adolescents and young adults, affecting ~5% of the population. Although an increased number of treatment options have become available, there is currently no cure for these conditions. Total enteral nutrition is an effective primary therapy for Crohn's disease, but the exact mechanism of action remains unspecified. The ability of CaSR agonists to reverse increased intestinal secretion and decreased absorption induced by bacterial enterotoxins, diminish overly active enteric nerve activity and motility, as well as restore compromised barrier function and imbalanced immune responses suggests that modulations of CaSR expression and activity using calcium mimetic or a combination of specific nutrients may provide a novel therapeutic approach for secretory diarrhea, IBS, and IBD.

## Author contributions

LT, CC, XS, AP, MM and SC contribute to the conception or design of the work, drafting or revising the manuscript, final approval of the version to be published, and agreement to be accountable for the content of the work.

## Funding

The National Institute of Health NICHD, award No. K08HD079674, the CDNHF/NASPGHAN foundation, award No. 00102979, and the Children's Miracle Network.

### Conflict of interest statement

The authors declare that the research was conducted in the absence of any commercial or financial relationships that could be construed as a potential conflict of interest.
