# Corrigendum: The Extracellular Calcium-Sensing Receptor in the Intestine: Evidence for Regulation of Colonic Absorption, Secretion, Motility, and Immunity

**DOI:** 10.3389/fphys.2016.00315

**Published:** 2016-07-19

**Authors:** Lieqi Tang, Catherine Y. Cheng, Xiangrong Sun, Alexandra J. Pedicone, Mansour Mohamadzadeh, Sam X. Cheng

**Affiliations:** ^1^Department of Pediatrics, Gastroenterology, Hepatology, and Nutrition, University of FloridaGainesville, FL, USA; ^2^Department of Medicine, Center for Inflammation and Mucosal Immunology, University of FloridaGainesville, FL, USA

**Keywords:** intestinal barrier function, enteric nervous system, gut immunity, secretory diarrhea, motility, irritable bowel syndrome, inflammatory bowel disease, calcium-sensing receptor

Due to an error in the original Review article, the name of the author Mansour Mohamadzadeh was incorrectly spelled as “Mansour Mahamadzadeh”. The authors apologize for the mistake and this error does not change the scientific conclusions of the article in any way.

The original article has been updated.

## Author contributions

All authors listed, have made substantial, direct and intellectual contribution to the work, and approved it for publication.

### Conflict of interest statement

The authors declare that the research was conducted in the absence of any commercial or financial relationships that could be construed as a potential conflict of interest.

